# Polyoxometalates (POMs) Memristors/Neuromorphic Devices: From Structure Engineering to Material and Function Integration

**DOI:** 10.3390/nano16070425

**Published:** 2026-03-31

**Authors:** Jufang Hu, Shengzhang Xu, Yanfang Meng

**Affiliations:** 1 Key Laboratory of Optoelectronic Devices and Systems of Ministry of Education and Guangdong Province, College of Physics and Optoelectronic Engineering, Shenzhen University, No. 3688, Nanhai Avenue, Nanshan District, Shenzhen 518060, China; jufanghuchemistry@outlook.com; 2 National Pipeline Eastern Crude Oil Storage and Transportation Co., Ltd., No. 1, Beijing Road, Quanshan District, Xuzhou 221116, China; 3 Department of Mechanical and Electronic Engineering, School of Mechanical Engineering, Jiangsu University, No. 301, Xuefu Road, Jingkou District, Zhenjiang 212013, China

**Keywords:** electronic features, polyoxometalate (POM), memristor, neuromorphic

## Abstract

The advancement of artificial intelligence and information technologies has presented higher demands on neuromorphic computing information devices, entailing the emergence of next-generation devices. Polyoxometalates (POMs) are emerging as promising molecular nanomaterials for next-generation neuromorphic computing, providing distinct advantages over conventional metal oxides. In contrast to bulk oxides that suffer from stochastic filament formation and device-to-device variability, POMs possess atomically precise structures with discrete, multi-electron redox states that enable highly reproducible and deterministic resistive switching. Their molecular nature allows for stable, multi-level data representation through stepwise reduction in metal centers (e.g., V, W, Mo) and the emulation of essential synaptic plasticity functions. Furthermore, the exceptional structural and chemical tunability of POMs favors covalent or supramolecular functionalization, enabling precise engineering of the POM-electrode interface and controlled self-assembly on surfaces. This molecular precision not only addresses the scalability challenges of traditional memristors but also unlocks unique functionalities, such as multimodal switching coupled with visible chromic response for state visualization. Taking the advantages of intermolecular crosstalk and countercation dynamics, POM-based networks offer a pathway toward constructing three-dimensional neuronal architectures, effectively connecting molecular redox chemistry to advanced high-density neuromorphic computing paradigms.

## 1. Introduction

Neuromorphic computing based on memristive devices presents a transformative paradigm to overcome the energy and speed limitations of conventional computing architectures [1,2,3,4,5]. A critical challenge for high-performance neuromorphic hardware is the demand for material systems capable of both stable analog switching and the rich physical dynamics needed to emulate complex synaptic functions [6,7,8,9,10,11]. Alternative material platforms, such as ferroelectrics, offer polarization-controlled resistance but face scalability and endurance hurdles at nanometer dimensions [12,13,14]. This landscape underscores a critical need for material systems that combine molecular precision, multiple accessible redox states, and structural tunability to enable deterministic switching and rich neuromorphic functionality [15,16]. Herein, polyoxometalates (POMs) emerge as a promising yet underexplored class of molecular materials for advanced memristive and neuromorphic applications. Their remarkable capabilities for molecular-level information storage stem from their intrinsic ability to accept and delocalize multiple electrons, which is essential for achieving high-density multibit storage [17,18,19,20,21,22,23,24]. Furthermore, POMs exhibit intrinsic multi-level switching capability, stemming from their propensity for multiple redox processes, during which electrons delocalize across the entire molecular framework, accompanied by tunable structural rearrangement [25,26]. By combining biomimetic temporal dynamics that mirror biological timescales [27,28,29,30] with controllable ionic crosstalk for functional interconnectivity [31,32,33,34], polyoxometalates (POMs) present a viable pathway toward more scalable and intelligent multi-level memory systems, holding the potential to revolutionize next-generation electronics [35]. The incorporation of multi-functionalized POMs platforms is being explored as a viable strategy for enhancing the scalability and intelligence of these systems.

This manuscript provides a critical analysis of the inherent properties of POMs to address specific challenges in memristor and neuromorphic device development. In Section 2, by contrasting the advantages, limitations, and existing challenges of other materials, we introduce POMs and systematically dissect their potential for neuromorphic devices based on three distinct structural characteristics, while also drawing a comparison with conventional metal oxides. Subsequently, we systematically analyze the mechanisms underlying POM-based devices and, through an in-depth investigation of influencing factors, explore structural design strategies (Section 3). Building on these approaches, we identify two characteristic advantages of POMs: their intrinsic multi-level resistive switching behavior and their tunable resistive switching at different interfaces (Section 4), as well as showcasing novel applications and fabrication techniques in Section 5. Finally, we present a balanced perspective on the outstanding challenges and future research directions for harnessing POMs in next-generation computing hardware (Section 6). Our summary aims to provide a coherent roadmap for researchers and engineers, illuminating how the unique molecular nature of POMs can be leveraged to design a new generation of high-performance, functionally rich neuromorphic devices.

## 2. Conventional Memristors and the Introduction of POM for Memristors

### 2.1. The Traditional Materials to Be Integrated: Memristors

#### 2.1.1. Metal Oxide Materials

**Figure 1:**
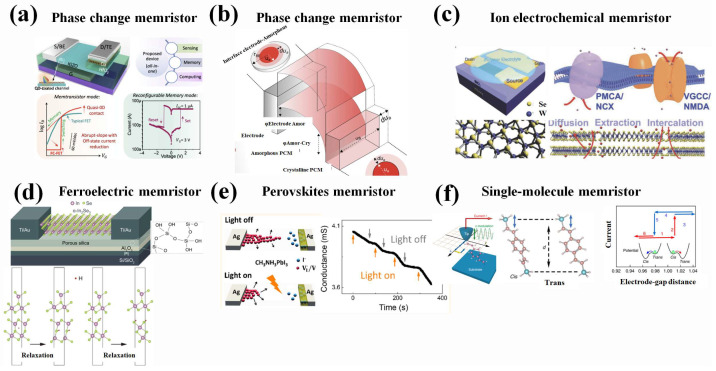
(**a**) (**Upper left)**: Schematic diagram of a device that features a single programmable unit enabling area- and energy-efficient, reduced-latency, fast-switching, multibit optic-neuromorphic system. (**Upper right**): A summary highlighting the integrated multifunctionality of the proposed device. (**Bottom left**) panel: The FC-FET demonstrates a filamentary conductive vertical transport with a resistive switching mechanism, where filament formation through the electrolyte layer governs the switching behavior. The V_G_ modulates the threshold switching, resulting in a near-ideal switching current transition facilitated by quasi-0D contact. The high resistance of the electrolyte layer in the off-state minimizes the device’s leakage or off-state current. Furthermore, the ability of filaments to form and rupture in a bias-dependent manner contributes to the realization of transistor-like memristor functionality. (**Bottom right**) panel: The I–V behavior under varying gate terminal E-fields (V_G_). The forming-free set operation transitions from HRS to LRS at a V_G_ of 3 V with a compliance current (I_cc_) of 1 μA, and negative sweeps lead to a sharp filament rupture during the reset operation [37]. Copyright: 2025 American Chemical Society. (**b**) A conceptual sketch of the energy-band profile after contact. The inset panels show the geometrical scaling of the two interfaces as a function of amorphous dome size (ua) [41]. Copyright: 2018 Wiley VCH. (**c**) (**Upper left**) panel: Schematic of the ion-gated synaptic transistor in this study, with van der Waals materials as the channel. (**Bottom left**) panel: Schematic illustration of WSe_2_ with hexagonal symmetry. (**Upper right**) panel: Dynamic balance between the Ca^2+^ entry of plasma membrane through voltage-gated Ca^2+^ channels or N-methyl-D-aspartate receptors and the Ca^2+^ extrusion via PMCA and NCX in biological systems. (**Bottom right**) panel: Field-directed Li^+^ migrations toward the channel and their extrusion via backward diffusions, similar to the dynamic processes in the upper right panel [44]. Copyright: 2018 Wiley VCH. (**d**) Device structure and working principle. Upper panel: Schematics of our three-terminal α-In_2_Se_3_ devices. Exfoliated α-In_2_Se_3_ flakes were placed on stacked hetero-structure gates, which were composed of Pt, Al_2_O_3_, and porous silica. Bottom panel: H atom (i) at the intralayer and (ii) at the vdWs gap [12]. Copyright: 2023 Science. (**e**) Left panel: Illustrations showing that light illumination can inhibit VI/VI × formation under bias (upper) and accelerate VI/VI × annihilation (lower). (**Right**) panel: Conductance of the device’s formation under bias (upper) and accelerated VI/VI × annihilation (lower) [55]. Copyright: 2018 American Chemical Society. (**f**) Left panel: Schematics of measurement setup (left) and tip control scheme. The STM tip, which was moved back and forth in the z direction in accordance with a sinusoidal function (green), was scanned two-dimensionally in the x (blue) and y (yellow) directions, as shown in the scheme. (**Middle**) panel: *Cis* and *trans* conformations of the DEB molecule formed in the Si/DEB molecule/Si junction. (**Right**) panel: Schematic showing the change in current [58]. Copyright: 2015 Nature.

Metal oxides represent the most fundamental and widely utilized material system for constructing memristors. Various oxides, including Gd_x_O_y_ [36], HfO_x_ [37], TiO_x_ [38], CuO_x_ [39], have been extensively explored. These devices primarily operate based on two dominant mechanisms: valence change mechanism (VCM) and electrochemical metallization (ECM) [40]. In VCM, resistive switching is driven by the migration of oxygen vacancies. Applying a voltage drives oxygen ions toward the anode, creating a localized chain of oxygen vacancies from the cathode. This vacancy-rich filament locally “dopes” the oxide, lowering the electronic barrier and enabling current flow (SET). A reverse voltage pushes oxygen ions back, re-oxidizing and breaking the path (RESET). The conductive state relies on a chemical change in the oxide’s stoichiometry. In ECM, an active metal oxidizes to form a conductive filament (SET) and dissolves under reverse bias (RESET), enabling resistive switching. Owing to their large switching ratios and high operational stability, metal oxide-based memristors have found widespread application in the field of artificial vision [37] (As shown in Figure 1a). However, significant challenges such as low device-to-device reproducibility and high forming voltages remain highly critical, constraining their further development and reliable large-scale integration.

#### 2.1.2. Phase-Change Materials

Phase-change memory (PCM) devices, predominantly based on chalcogenide glasses (e.g., GeSbTe) and select metal oxides (e.g., VO_2_), operate through reversible phase transitions between amorphous (long-range disorder) and crystalline (long-range order) states. The transition from crystalline to amorphous state is achieved by applying a heat pulse that elevates the temperature above the melting point, followed by rapid quenching. These devices offer advantages such as remarkably low switching energy (ranging from hundreds of picojoules to a few nanojoules) and ultrafast switching dynamics at nanosecond timescales [41]. However, despite these merits, their inherent granular or layered structures introduce significant device-to-device variations, posing substantial challenges for large-scale integration and uniform performance, as illustrated in Figure 1b.

#### 2.1.3. Ion Electrochemical Materials

Addressing the drawbacks of the above two types of memristors, ion electronics devices have been proposed as alternatives with uniform and deterministic control of electronic conductivity and relatively low energy consumption. The main mechanism involves trap trapping mechanism [42], the ion intercalation mechanism [43,44], the phase transition mechanism [45,46], and the ion regulation mechanism [47]. During the writing process of the device, ions are transported through an electrolyte that separates the gate from the channel while maintaining corresponding electron flow in the external circuit. The nonvolatile characteristics arise from the immobilization of ions in the channel when the gate/reservoir is electrically disconnected, preventing electron flow and maintaining the programmed state. The performance characteristics are significantly influenced by the intrinsic properties of the ionic species, selection of gate/channel/electrolyte materials, and fabrication process temperatures (As shown in Figure 1c) [42]. Particularly, their unique CMOS compatibility facilitates integration with silicon-based deep learning accelerators. However, the adoption of ion-based memristors remains constrained by their low switching speeds, which largely originate from inherent ionic relaxation dynamics [48].

#### 2.1.4. The Memristors Based on Ferroelectric-Based Materials

To address the limitations of phase-change memories (PCMs), ferroelectric materials memristors have followed the fashion. The working principle is that when the electric field is above the threshold, the intrinsic ferroelectric characteristic drives the electric dipole aligned between an upward and downward direction switch. Recently, the incorporation of ferroelectricity into the array structure to achieve high-performance, high-density neural networks has become a research hotspot [49,50,51]. Application of progressively increasing voltage pulses to the gate electrode induces gradual polarization reorientation toward the upward configuration, enabling precise modulation of channel conductance through progressive polarization switching under incremental pulse stimulation (As shown in Figure 1d) [12,52]. Despite fabrication complexities and stringent process controls, along with material stability, sensitivity, and reliability issues, continued R&D is crucial to optimize ferroelectric memristors for practical applications.

#### 2.1.5. Perovskite Materials

Despite significant advancements achieved in memristor technologies based on phase-change materials, ferroelectric materials, and ion-intercalation systems, persistent challenges remain regarding device-to-device uniformity, energy efficiency, and limitations in further performance enhancement. The emergence of perovskites-based memristors has emerged as a superior candidate compared to other memristors, owing to their tunable bandgap, delicate lattice structure, high defect tolerability, and solution processability [53]. The working principle of perovskite memristors fundamentally involves the ion migration mechanism [54,55] (As shown in Figure 1e). However, due to their poor solvent resistance, perovskite materials face significant challenges in achieving long-term stability (over five years), which severely limits their practical application.

#### 2.1.6. The Memristors Based on Single-Molecule Material

To solve the shortcomings of undesired controllability of perovskites-based memristors, single-molecule non-volatile memories can be proven as high-density memristors with in-memory computing capabilities for processing real-time data, which show the prospects to surpass the limitations predicted by the Von Neumann architecture. Single-molecule memory devices achieve non-volatile resistive switching through multiple controllable mechanisms that enable bistable or multistate operation, which enables them to realize multistable conductance states, such as charge changes, conformational transformations, tautomerisms, intramolecular motions, and spin changes. The transformation between bistable structures is sensitive to thermal relaxation. As a result, the transformation of a bistable state needs an appropriate barrier [56,57,58,59] (As shown in Figure 1f).

### 2.2. POM-Based Memristors

Polyoxometalate (POM)-based memristors represent a convergent interdisciplinary platform combining materials chemistry and nanoelectronics [60,61]. POMs (known as iso-poly-anions and hetero-polyanions) are nanometer size metal oxide clusters exhibiting considerable variety in charge and framework structures. They can be expressed by the general formula:

[M_m_O_y_]^n−^   Isopolyanions

[X_a_M_m_O_y_]^n−^  Heteropolyanions

#### 2.2.1. Introduction and Classification of Polyoxometalates (POMs)

POMs-based memristors or neuromorphic devices are geometry-dependent with foundational three categories: the Keggin type-POM, Dawson type-POM and the Lindquist-type. The combination of abundant anions and redox-active species with the electron-accepting capabilities of polyoxometalates (POMs) enables unique functionality. Notably, both Keggin and Dawson structural archetypes can undergo controlled degradation under specific conditions to generate lacunary POM anions. Keggin-type POMs require much greater efficiency in power and processing speed for massive computational tasks, such as pattern recognition and classification. For decades, research in polyoxometalate (POM) chemistry has predominantly focused on Group 6 metals (Mo and W). While studies of Group 5 vanadium-oxo clusters progressed more slowly, they nevertheless advanced significantly beyond their niobium and tantalum counterparts. Since the 21st century, structural investigations of polymolybdate/tungstate systems have emphasized two major directions: (1) the design of giant polymetal–oxo clusters [62,63,64,65,66] and (2) the engineering of sandwich-type architectures [67,68,69].

In contrast, the incorporation of organic components into polyoxometalate (POM) frameworks yields novel organic–inorganic hybrid materials with enriched structural diversity. These hybrid compounds exhibit synergistic properties derived from both their inorganic POM cores and organic functional groups. These hybrid systems exhibit integrated preformation arising from both covalent and non-covalent interactions between components. The non-covalent interactions include electrostatic forces, hydrogen bonding, van der Waals forces, and hydrophobic effects. Structurally, POM-based organic–inorganic hybrids can be classified into two categories: (1) Material systems are primarily categorized into two major classes based on compositional complexity: binary systems, defined by the integration of two functional groups or components, and ternary systems, which constitute more sophisticated architectures by incorporating three distinct functional elements [70,71,72]. In a two-component system with a polyoxometallic anion and an organic cation, the organic part plays a role in charge balance, space filling, structure orientation, or is directly coordinately attached to a polymetallic skeleton. POMs, as a unique class of anionic metal-oxo clusters, readily form organic–inorganic hybrid compounds through association with organic countercations (e.g., protonated amines or ammonium derivatives) via various non-covalent interactions. The organic part can also be introduced by substituting the coordination oxygen atom of the metal oxygen within the metal–oxygen cluster skeleton, generating a series of organically functionalized POMs with organic nitrogen, organosilicon, organophosphorus substituted organic functionalized polymetal–oxygen clusters, and organic–inorganic hybrid compounds, which are known as covalently bonded polymetallic oxygen cluster compounds [73,74].

Precision engineering of polyoxometalate (POM) architectures for storage medium through covalent/noncovalent functionalization represents the most effective strategy for developing high-performance memory. Polyoxometalates (POMs) exhibit two defining characteristics: fine-tuning redox and high ability to accept electrons with negligible structural transformation. As a result, accepted electrons can be delocalized throughout their metal–oxygen framework. The above characteristic offers a platform for application in electronic devices [75,76,77,78]. Electronic properties of the three types of POMs are systematically analyzed and conclude that the Keggin-type POMs are preferred for engineering single-molecule electronic devices, Dawson type POMs are feasible for constructing MOS flash memories, while Lindquist-type POMs are suitable for application to ultra-high-density memory devices.

(a) Keggin type

The most used metals of Keggin-type POMs are Mo with the highest oxidation state of +6 [79,80]. Typically, switchable and reversible electron-transfer processes between metal centers make Keggin-type POM the desired candidate for engineering of single-molecule electronic devices [81]. Keggin type-POM prefers to be applied as a redox-based nonvolatile memristor, where the nanoscale redox process is attributed to resistance change under an external electrical stimulus, demonstrating great potential for application in next-generation memory and neuromorphic computing systems. To address the limitations of bulk transition metal oxide materials employed in current redox-based resistive switching memory, POMs show high suitability for the next-generation molecular electronic devices for their particular advantages, nanoscale size, high stability, and rich reversible redox potential. Similar to transition metal-oxides, intrinsic migration of oxygen ions can also be responsible for the main resistance to the redox property of POMs [82].

(b) Dawson-type

Besides the generally studied Keggin POMs memristor, Dawson type POM clusters are also feasible to be incorporated into memristors or devices. The most widely used metals of Dawson-type POMs are Mo and Se with the highest oxidation states of +6. The low binding energy between metal centers and low electron dissociation energy make them liable to redox reaction, and subsequently, resistance change occurs. The {(SO_3_)_2_Mo_18_O_54_} construct is integrated into an integral intramolecular configuration by two embedded pyramidal sulfite redox agents. Particularly, {Mo_18_O_54_} oxide shell transition from {Mo^VI^_18_} with a totally oxidized state at 77 K to {Mo^VI^_16_Mo^V^_2_} and a mixed valence state at 298 K. Furthermore, this reversible electron transfer also conforms to the formation of a bond between the two encapsulated pyramidalsulfite (SIVO_3_)_2_ groups at 298 K. The local electric field of adsorbing the POM cluster on the gold surface facilitates this redox behavior. Consequently, the formation of the S-S bond inside the {Mo_18_O_54_} cluster shell could produce a new populated electronic state in the bandgap of the{(SO_3_)_2_Mo_18_O_54_} cluster [83]. Taking advantage of their tailorable electronic configuration, Dawson type POMs have been applied as high-density storage nodes for flash memories. The performance of the {Mo_18_O_54_(SO_3_)_2_}^4−^-based device is closely related to the position of the POM molecules integrated into the storage media. Comparative investigation of the electrical behavior of the core–shell POMs with central cores as [(Se(IV)O_3_)_2_]^4−^ (where selenium exists as Se^4+^) and [Se(V)_2_O_6_]^2−^ (selenium exists as Se^5+^) revealed that the emergence of higher oxidation state could be employed to design a novel “write once–erase” memory.

(c) Lindqvist-type

Polyoxometalates (POMs), particularly vanadium-based variants (V-POMs) such as the Lindqvist-type hexavanadate (V6), represent a promising class of molecular materials for neuromorphic computing due to their unique redox-driven resistive switching behavior [25,84]. These inorganic–organic hybrids exhibit remarkable reliability through atomically well-defined structures and high thermal/chemical stability, enabling reproducible multi-level switching—as demonstrated by STM studies showing V6 functions as a single-molecule 4-level switch at room temperature [27,85]. This volatile multistate resistance modulation, governed by stepwise reduction in vanadium centers and accompanied by countercation dynamics, directly supports essential neuromorphic functions including short-term memory (STM) with biologically relevant timescales of 50–300 ms [28], long-term potentiation through controlled ion diffusion [29,30], and the emulation of synaptic plasticity [31,32]. Furthermore, the inherent capacitive behavior arising from ion compensation mechanisms mirrors neuronal ion transport, positioning V-POMs for in-memory computing paradigms such as processing-in-memory (PIM) [33]. By exploiting intermolecular crosstalk via mobile countercations rather than suppressing it—a key distinction from classical memory applications—V-POM networks can potentially implement self-training processes and pattern classification through collective ionic interactions [34]. The strategic immobilization of either POMs (via DNA origami, carbon nanotubes, or polymer matrices) or their countercations (using bulky organic cations) enables the construction of three-dimensional neuronal networks capable of sophisticated information processing, bridging molecular redox chemistry with neuromorphic computing architectures.

#### 2.2.2. A Comparison of the Three Structural Classes of Polyoxometalates

Polyoxometalates (POMs) exhibit distinctive structural characteristics among their three archetypal forms, which essentially govern their redox behavior (Table 1). The feature of Dawson-type POMs lies in two embedded redox agents capable of reversible interconversion between electronic states. When thermal activation occurs, two electrons are expelled from the sulphite anions and delocalized over the metal oxide cage, transitioning the cluster from a fully oxidized state to a two-electron reduced state, concomitant with the formation of an S–S bond between the two sulfur centers. This behavior contrasts with Keggin- and Lindquist-type POMs, which possess shorter average bond lengths, higher bonding energies, and greater resistance to redox processes—a direct consequence of their internal molecular architectures. Bond length distribution analyses uncover trimodal patterns in Keggin molybdates: a symmetric population centered at 1.92 Å alongside nonsymmetric contributions at 1.85 and 2.00 Å. The incorporation of anions of group IVA heteroatoms exhibits exclusively bimodal distributions, indicating that increased negative charge of the internal XO_4_q^−^ unit favors distorted configurations, while lower-charge anions may adopt either symmetric or nonsymmetric forms.

As a next step, following our exploration of the operational mechanisms of POM-based memristors, we will conduct a comparative analysis between these devices and the most widely used metal oxide memristors.

## 3. The Mechanism of POMs for Memristor/Neuromorphic Device

### 3.1. Mechanism of POM-Based Memristors

Fully oxidized polyoxometalate (POM) molecules exhibit remarkable structural stability in both solid and solution phases, maintained by charge-balancing countercations that neutralize their anionic frameworks. Generally, the highest occupied molecular orbitals (HOMO) display oxygen p- orbital character (oxo band), and the set of lowest unoccupied orbitals (LUMO) predominantly display metal d- orbital character (metal band) [76]. Notably, though these sets of orbitals do not form a band in the conventional sense, the orbital energy is separated by discrete energies in these medium-sized clusters [86]. In the classic Wells–Dawson clusters with a cylinder shape, the HOMO demonstrates predominant delocalization across oxygen atoms, while the non-classic clusters with a hourglass-shape is delocalized over the [SO_3_]^2−^ moiety. Remarkably, in both structural paradigms, the subsequent molecular orbitals remain localized on the equatorial metal centers. As a consequence, in both cases, the LUMO is delocalized over metal centers that are connected to each other by large M-O-M angles [87] with a narrow value range. Under a voltage, the first reduction takes place in the equatorial (belt) region, yet reduction in the cluster does not alter the net charge on the internal heteroatoms, because the additional electrons go to addenda symmetry-adapted orbitals [88,89].

#### 3.1.1. Electrochemical Metallization Mechanism

**Figure 2:**
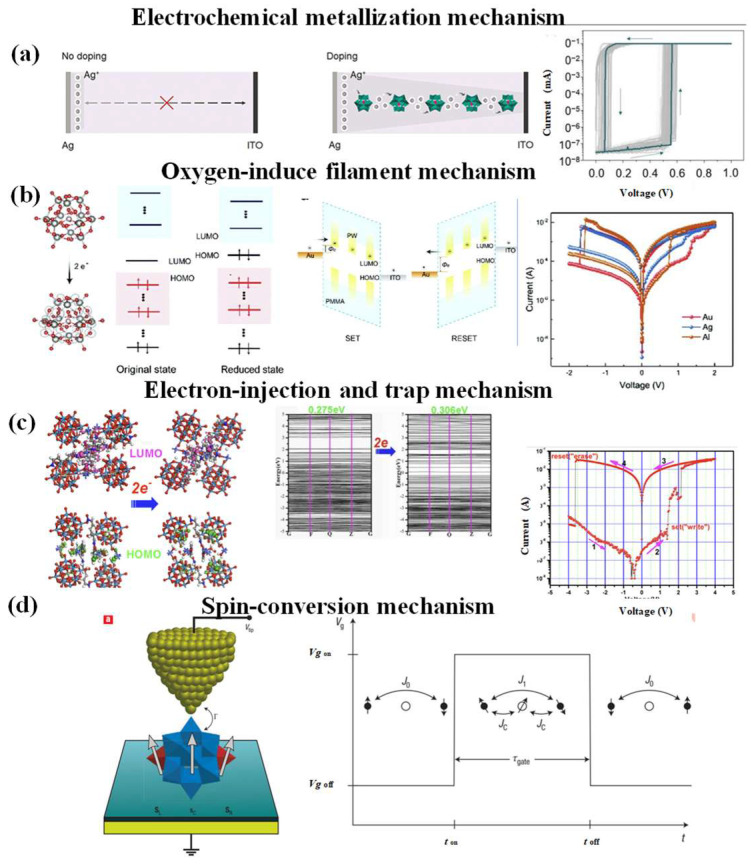
(**a**) (**Left**) panel: Schematic comparison of the Ag migration in pristine P3HT and P3HT@TDA-PW. (**Right**) panel: Current–voltage (I–V) curve of the device during 300 cycles [30]. Copyright: 2022 Wiley VCH (**b**) (**Left**) panel: Frontier orbitals depicting the charge density states of the HOMO and LUMO of the PW cluster before and after two electrons were accepted, obtained by DFT calculation. (**Middle**) panel: The schematic illustration of interface barrier modulation during the SET and RESET processes. (**Right**) panel: I–V characteristics of the memory device with Au, Ag or Al as the top electrode [81]. Copyright: 2019 Royal Society of Chemistry. (**c**) (**Left**) panel: electron-density distributions of the HOMO and LUMO before and after accepting two electrons. (**Middle**) panel: Conduction mechanism of the ITO/POMOF/Ag device at room temperature. (**Right**) panel: I–V curves of the ITO/POMOF/Ag device with one cycle [90]. Copyright 2021 American Chemical Society (**d**) (**Left**) panel: Schematic drawing of the polyoxometalate {PMo_12_O_40_(VO)_2_}^q−^ separated by an insulating tunneling barrier from a metallic lead and contacted via a tunnel coupling G to a tip at a potential Vtip. Indicated are the two localized spins SL and SR of the red VO_5_ square pyramids. Depending on the redox state of the molecule, the delocalized valence electrons of the blue MoO_6_ octahedra form a spin sC or pair to form a singlet, enabling two-qubit electrical gating. (**Right**) panel: Quantum-gate sequence for the pSWAP operation and exchange coupling constants during the corresponding gate phase [80]. Copyright: 2007 Nature.

The resistive switching mechanism regarding electrochemical metallization and the mechanism of POM-based devices (H_3_PW_12_O_40_) involves the coordinated migration of oxygen ions and the generation of oxygen vacancies. During the positive bias sweeping (SET process), the reduction (for example, the electron-trapping process at the interface) and the transportation of generated charge carriers between H_3_PW_12_O_40_ clusters conclusively dominated the conduction mechanism, resulting in progressive resistance switching behaviors (As shown in Figure 2a) [30].

#### 3.1.2. Oxygen-Induced Filament

The field-induced redox of POM ligand oxygen atoms and oxygen vacancy migration form an essential resistive switching mechanism, as demonstrated by both frontier orbital (HOMO/LUMO) theory and experiments [81] (As shown in Figure 2b). The frontier orbital (HOMO/LUMO) theory points out that barrier modulation aligns with the established role of interfacial charge trapping in altering the adjacent Schottky barrier at the metal–oxide interface [91], providing a consistent electronic rationale for the observed switching asymmetry between the SET and RESET processes. During the SET process, the resistive transition proceeds progressively and is governed by two sequential mechanisms: first, the reduction in PW molecules (e.g., electron trapping at the interface), followed by inter-molecular charge transport between neighboring POM clusters. In contrast, the RESET process exhibits a more abrupt transition from the low-resistance state (LRS) to the high-resistance state (HRS), attributable to enhanced electron extraction and recombination efficiency that facilitates rapid charge dissipation—a phenomenon well-documented in prior studies. Collectively, these results indicate that the reduction in the POM cluster initiates the resistive switching sequence. A rational mechanistic pathway is that electron injection into POM molecules via Fowler–Nordheim tunneling across the Au/PW interface, followed by charge hopping between POM molecules through energetically favorable sites (As shown in Figure 2b). This process concurrently modulates both the bulk resistance of the active layer and the height of the interfacial Schottky barrier, thereby governing the overall memristive response [92,93,94].

#### 3.1.3. Electron-Injection and Trap

**Figure 3:**
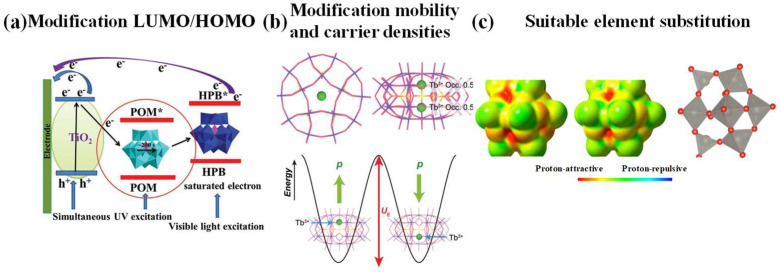
The strategies to increase ON/OFF (**a**) Schematic representation of electron transfer in this electrode [96]. Copyright 2018 Wiley VCH. (**b**) Top panel: Side and top views of [Tb^3+^ P5W_30_O_110_]^12−^ ([Tb^3+^ ⊂ P5W_30_]) cluster. [Color code: green (Tb), red (O), purple (W), and yellow (P)]. (**Bottom**) panel: Schematic showing the double-well potential structure of the dipole moment with energy barrier UE between two stable terbium sites in [Tb^3+^ P5W_30_O_110_]^12−^. The direction of the dipole moment (p) is determined by the localisation site of the terbium ion [97]. Copyright 2018 Wiley VCH. (**c**) MEP mappings in β-PMo12, β-PW12, and the pristine model of β-PM12. Reddish and bluish regions in MEP mappings represent proton-attractive and repulsive regions [98]. Copyright 2024 Royal Society of Chemistry.

To directly probe the electronic nature of the POMOF during resistive switching, density functional theory (DFT) calculations were performed using the CASTEP program (As shown in Figure 2c) [90,95]. This enables visualization of the evolution of electron occupancy before and after the cluster accepts two electrons, with the corresponding band structures and electron-density distributions of the frontier molecular orbitals (HOMO and LUMO) presented in Figure 3c middle upper panel and Figure 2c middle upper panel. The band gap shows only a minor change upon the injection of two electrons (from 0.275 eV to 0.306 eV). This subtle increase aligns with the device’s high-resistance state (HRS) at low bias, reflecting an enhanced electron injection barrier. The more significant transformation, however, occurs in the composition of the frontier orbitals. In the initial (oxidized) state, the HOMO primarily consists of π-bonding orbitals from the ligand, mixed with a minor contribution from the O-2p orbitals of the POM, while the LUMO is characterized by π* orbitals of the ligand alongside the d-orbitals of Co ions. After accepting two electrons, the HOMO becomes dominated by O^b^/O^c^-2p orbitals localized on the POM, and the LUMO exclusively comprises the π* orbitals of the ligand. This marked shift in orbital character strongly indicates that the injected electrons are primarily accepted and stabilized by the POM cluster, a process facilitated by the coordinated metalloviologen framework.

#### 3.1.4. Spin-Conversion Mechanism

Spin conversion mechanism also exists within the POM with a spin-dependent electron configuration [19,80,99]. For example, Loss et al. [80] propose an experimental design for implementing a molecule-scale all-electric two-qubit gate and readout system using {PMo_12_O_40_(VO)_2_}^q−^ (As shown in Figure 2d). Such a polyoxometalate consists of a central mixed-valence core based on the [Pmo_12_o_40_] Keggin unit, which is capped by two vanadyl groups containing two localized spins. The Keggin core functions as a tunable electron reservoir, where delocalized electrons can migrate across molybdenum centers and mediate weak magnetic coupling between the two (VO)^2+^ units. For example, when the mixed-valence Keggin core hosts an even number of electrons, the spins pair in an anti-ferromagnetic style to form a total spin 0 state. The system involves the two spins S = 1/2 on the vanadyl groups, weakly coupled by means of an indirect exchange mechanism mediated by the core electrons. When the number of core electrons is odd, an unpaired spin 1/2 on the core remains, and one obtains a set of three coupled spins 1/2. This magnetic coupling can be switched in an all-electrical fashion and can be used for the implementation of a fundamental two-qubit gate by modifying the occupation electron number of the central core in the {PMo_12_O_40_(VO)_2_}^q−^ cluster via tuning the gate voltage Vg, which corresponds to the different redox potentials of the cluster. The magnetic coupling in the {PMo_12_O_40_(VO)_2_}^q−^ system can be electrically modulated through gate voltage (Vg) tuning, which alters the electron occupation number of the Keggin core via its redox-active properties. This enables all-electrical control of a fundamental two-qubit gate operation, where distinct redox states correspond to specific coupling regimes. Mialane et al. conducted systematic investigations in the structural and magnetic properties of Mn-III and Cu-II Tetranuclear azido polyoxometalate complexes [19]. The working principle of POM-based electronic devices is generally not confined to a single mechanism but often involves a combination of several. By strategically modulating the charge stabilization of reduced POMs through chemical functionalization, countercation selection, surface engineering, and intermolecular interactions, diverse physical switching mechanisms can be precisely engineered. This tunability underscores the remarkable uniqueness and design versatility of these molecular oxides [29].

### 3.2. Factors Determining the Performance of POM-Based Memory

Polyoxometalates (POMs) demonstrate exceptional charge-trapping capabilities that fulfill the critical requirements for next-generation neuromorphic computing systems. Owing to the capabilities of multiple discrete redox states in a narrow potential range, POMs demonstrate suitability for designing a high-performance rewriteable multi-level resistive memory [100]. Beyond charge-trapping and oxygen vacancy migration, the memristive functionality of POMs arises from their intrinsic ability to tune HOMO–LUMO gaps and adjust charging levels via modulated electron localization, all while maintaining operational stability at room temperature [70,85,101,102].

The performance parameters typically used to characterize traditional memristors—such as ON/OFF ratio, storage ability, stability, speed, and others—are also applicable to POM-based memristors.

#### 3.2.1. ON/OFF

**Figure 4:**
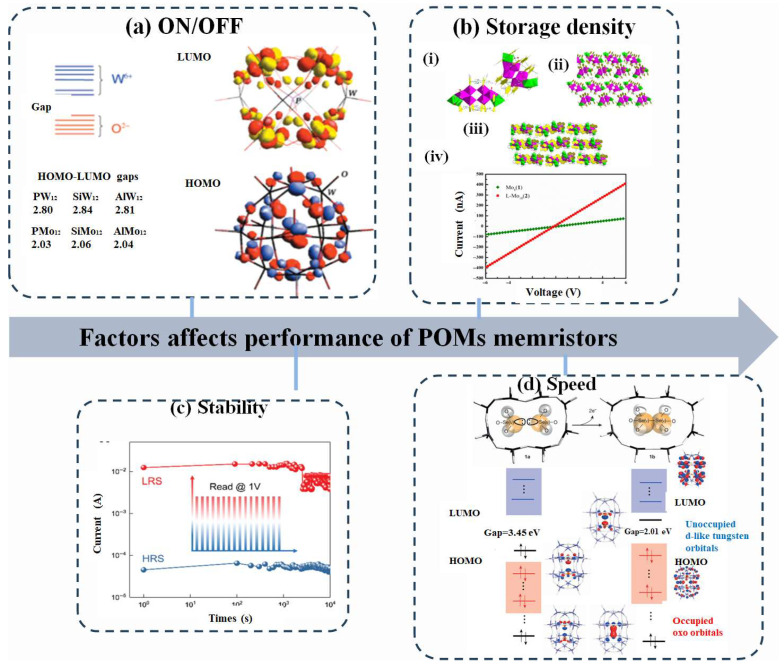
(**a**) Schematic orbital diagram, HOMO–LUMO energy gaps (in eV) and 3D representations of one of the two doubly degenerate components of the LUMO and HOMO, the fully oxidized [PW_12_O_40_]^3−^ anion [104]. Copyright: 2001 American Chemical Society. (**b**) Upper panel: (**i**) Intramolecular face-to-face and intermolecular C−H⋯π stacking interactions in L-Mo10 (2) (Å) between 2,2′bipyridine. (**ii**) 2-D π⋯π stacked layer though weak interchain C−H⋯π interactions (3.804 Å). (**iii**) 3-D π-stacking supramolecular structure through weak interlayer C−H⋯π interactions (3.995 Å); color code: Mo^IV^, pink; Mo^V^, green; O, red; S, yellow; C, gray; N, blue; H, sky blue. (**iv**): I–V characteristics of Mo8 (1) (green) and L-Mo10 (2) (red) [105]. Copyright: 2024 American Chemical Society. (**c**) Retention performance in the LRS and HRS when read at 1 V. (Inserted) Double logarithmic plots of the I–V curves of the ITO/PW@PMMA/Au memory device during the SET process [81]. Copyright 2019 Royal Society of Chemistry. (**d**) Scheme depicting the formation of the Se(V)–Se(V) bond within the cluster cage. At the top, a schematic diagram shows the formation of the Se(V)–Se(V) bond in the transformation of 1a to 1b. At the bottom are the results from the DFT analysis, demonstrating the frontier orbitals and the formation of the Se(V)–Se(V) bond. Relevant orbitals delocalized over the Se moieties are highlighted in bold. The HOMO–LUMO gap is the energy gap between the highest occupied molecular orbital (HOMO) and the lowest unoccupied molecular orbital (LUMO). Although the orbital energies of POM clusters are separated by discrete energies, they can also be viewed as having a pseudoband-like orbital structure, and in this sense, the blue box depicts the set of unoccupied tungsten d-like orbitals and the red box the set of occupied oxygen p-like orbitals [106]. Copyright: 2008 Nature.

ON/OFF, the ratio of the current value in the low resistive state to the current value in the high resistive state, represents a key parameter in a neuromorphic device. A large portion of memories confront the challenges of low ON/OFF ratios. A single POM molecule typically exhibits a limited ability to reversibly accept/release electrons. Furthermore, charge transport in POM-based systems still relies on intermolecular electron hopping, which inherently restricts carrier mobility and results in a low switching ratio (As shown in Figure 4a) [103,104].

##### Strategies to Increase ON/OFF

We analyze the approaches to improving the on/off ratio from a fundamental perspective. Device Turn-On: When the device turns on, charge is injected from the electrode into the material. To make I_ON_ as large as possible, the Fermi level (work function) of the electrode should be precisely matched with the material’s LUMO (in the case of electron injection, n-type transport) or HOMO (in the case of hole injection, p-type transport). If the energy levels do not match, a higher injection barrier forms, resulting in a lower I_ON_. Therefore, the specific positions of the LUMO or HOMO energy levels are crucial for achieving high turn-on conductivity. Charge Transport: After injection, charge transport within the material relies on transport channels within the LUMO or HOMO energy levels. High mobility is necessary for high I_ON_, and mobility, in turn, is related to the material’s molecular packing, energetic distribution, and energy level alignment [107,108]. Consequently, the LUMO/HOMO levels and the mobility and carrier densities are the main factors influencing the on/off ratio.

1. The modification of LUMO/HOMO

The positional deviation between the width of the LUMO level of the POMs and the EF of the metal electrode determines the electron injection barrier and electron injection process, further posing impact on the performance of the memristors. The position of the LUMO level of molecular POMs is usually below the EF of the metal electrode; as a result, electron transfer from the metal electrode to POMs [96,109]. The HOMO and LUMO levels, dictated by the central atom’s element, in turn dictate the cluster’s fundamental redox behavior. Frontier molecular orbital engineering through heteroatom substitution (e.g., W/Mo replaced by V) or heteroanion incorporation effectively tunes POM electronic configurations, optimizing redox properties for synaptic emulation [96] (As shown in Figure 3a).

2. The modification of mobility and carrier densities

Based on the XPS and electrochemical analyses in this work, the proposed electron transfer pathway was validated through experimental characterization rather than DFT calculations. The shift and splitting of W4f_7_/_2_ XPS signals (35.8 eV and 36.5 eV) confirmed environmental changes around W centers upon POM immobilization, directly correlating with altered redox behavior. Cyclic voltammetry further supported this by revealing modified reduction waves (−1.6 V) for immobilized POMs compared to solution-phase analogs (−1.1 V), consistent with electrostatic interactions and restricted electron transfer through the organic underlayer. These experimental observations collectively substantiate the hypothesized switching mechanism involving countercation-mediated charge compensation [110,111].

At elevated temperatures, the Tb^3+^ ions in Tb-based POM exhibit thermally activated hopping behavior between the two available coordination sites within the {P_5_W_30_O_110_} polyoxometalate framework. Additionally, the hopping and subsequent localization of Tb^3+^ ions modulate local charge distribution and carrier densities, thereby influencing the resistive switching behavior. This ionic motion enhances the mobility of charge carriers and contributes to multi-level conductance states without inducing structural phase transitions. The resulting ferroelectric-like behavior, driven by localized dipole realignment rather than long-range ordering, supports non-volatile memory functions and facilitates synaptic plasticity in neuromorphic applications [97] (As shown in Figure 3b). The relationship between carrier densities and the energy barrier was revealed through dielectric measurements, helping to elucidate the underlying charge transport mechanism.

3. Atomic-scale structural parameters (especially protonation behavior)

The electron delocalization can be systematically modulated by some chemical and structural perturbations. For example, the incorporation of substitutes into the metal–oxygen framework disrupts the equivalence of neighboring metal centers and/or alters orbital hybridization through bridging atoms. DFT calculations have elucidated the pivotal role of these heteroatoms for the electron transfer in doubly reduced polyoxometalates, inducing a transition from delocalization-mediated spin-paired states to conventional antiferromagnetic coupling (or ferromagnetic) exchange, which demonstrates significant electronic structure modifications during redox processes [112]. DFT calculations were performed on Keggin-type polyoxometalates (POMs) to verify protonation behavior governing resistive switching. The computations identified that protonation sites are determined by addenda metal species and total cluster charge, with oxygen atoms exhibiting greater negative charge serving as preferential protonation sites. Crucially, the first protonation energy is determined by total charge and the core–shell bond length (O_h_–M), establishing a direct link between atomic-scale structure and proton-coupled electron transfer. These structural parameters correlate strongly with experimentally observed redox switching, where preferential protonation at specific oxygen sites facilitates reversible electron transfer between metal centers (Mo^6+^/Mo^5+^ or W^6+^/W^5+^), manifesting as multi-level resistive switching. (As shown in Figure 3c) [98].

#### 3.2.2. Storage Density

The memristor based on POM generally suffers from low electrical conductivity and high resistance, which restricts its practical implementation in CMOS technologies. Despite great progress having been made to explore advanced molecule-based flash memory, several fundamental challenges persist that impede its practical implementation for conventional CMOS technologies [70].

DFT calculations verify that stronger intramolecular face-to-face π stacking interactions, which are interconnected by intermolecular C−H⋯π into a three-dimensional framework structure, are responsible for highly enhanced electronic conductivities of Mo^IV^_6_(bpy)_6_Mo^V^_I2_O_16_S_2_]·10H_2_O. DFT calculations computed the electronic structures, including HOMO–LUMO gaps (2.56–2.63 eV), frontier orbital compositions, and electrostatic potential maps. The calculations assumed periodic boundary conditions for crystalline models and employed GGA-PBE functionals. Results revealed that HOMOs comprise Mo^IV^–Mo^IV^ bonding orbitals from superelectron-rich [Mo^IV^_3_O_5_S] units, while LUMOs consist of bpy π* orbitals. This electronic configuration links directly to the experimentally observed switching behavior: photogenerated electrons can transfer from occupied Mo–Mo bonding orbitals to vacant bpy π* orbitals via π-stacking pathways, enabling the multi-level conductance states essential for memristive switching. The DFT-calculated energy level alignment thus rationalizes the experimentally determined conductivity enhancement and provides a mechanistic basis for the resistive switching mechanism [105] (As shown in Figure 4b). To address the lower high storage density of major POM, Christoph Busche et al. 29 demonstrated a high-density polyoxometalate (POM)-based memristor architecture that exhibits unparalleled promise for next-generation CMOS-compatible flash memory applications. The exceptional storage density of this polyoxometalate (POM)-based memory device originates from the intrinsic properties of the core–shell POM clusters, at both the molecular and device level, that embedding [(Se(IV)O_3_)_2_]^4−^ as an oxidizable dopant in the cluster core allows the oxidation of the molecule to a [Se(V)_2_O_6_]^2−^ moiety containing a{Se(V)–Se(V)}bond (where curly brackets indicate a moiety, not a molecule) and reveals a new 5+ oxidation state for selenium.

(a) Suitable dopant substitution

The suitable metal complex is proven to be a versatile and effective strategy for tailoring the conductivity. However, a challenge exists in developing piezoelectric ceramics with high conductivity merely through combining the metal ion doping substitution and the typical sintering procedure. One-dimensional superlattices constructed from alternating POMs and Ag clusters exhibit enhanced electron transfer kinetics and superior performance compared to three-dimensional counterparts, owing to their interactive electronic structures. However, challenges remain in scalable synthesis, precise alignment control over long ranges, and long-term operational stability of the Ag–POM interface. Additionally, understanding the structure–property relationships governing device-to-device variability is critical for practical integration into reliable memory and neuromorphic architectures [113].

(b) Layer structure

In addition, POM can increase their storage density by engineering their packing structure [100,114,115]. The structural characteristic of POMs is crucial for the electron transport. Previous studies have uncovered that at relatively lower voltage, the two mechanisms, percolation and tunneling, coexist, which is conclusively demonstrated by the consistent linearity observed in all the Fowler–Nordheim(FN) curves (1/V^2^)~(1/V), while at high voltage bias, FN tunneling plays a dominant role (as indicated by the exponential dependence of 1/V^2^ over 1/V) in excluding POM-ending films with a 150 nm gap width. The onset of FN tunneling for different samples proceeds at different biases; the lowest, the intermediate and the highest ones corresponded to type A (50 nm gap width, POM-ending film), type D (50 nm gap width, DD-ending film), and type E (150 nm gap width, DD-ending film), respectively [100].

When POM materials are in direct contact with the semiconductor layer, the energy level alignment occurs between the conduction band of the semiconductor and the LUMO of the POM material, creating highly efficient electron injection, which fulfills performance requirements for flash memory applications.

For instance, theoretical calculations reveal that the HOMO and LUMO of the [W_18_O_54_(SO_3_)_2_]^4−^ clusters align below the conduction band of Si. Furthermore, due to the extra electron demanded upon the reduction in the POM cluster, the spatial localization of the HOMO and LUMO of the [W_18_O_54_(SO_3_)_2_]^4−^ clusters is caused by the changes in electron density with different redox states. Consequently, [W_18_O_54_(SO_3_)_2_]^4−^ clusters can act as mono-energetic trap centers [115].

(c) Adding the second unit

Through incorporating reduced POM/Al cathode interfaces, the operational characteristics of electronic devices have been considerably improved, which contributed to a considerable decrement of the electron injection/extraction barrier and an enhancement of electron transport and the reduced recombination losses in our reduced POM modified devices [15].

(d) Modifying with polymer

The introduction of external polymers provides the POM multi-level resistive switching memory properties, significantly enhancing the data storage density of flexible electronic devices. For example, with 1,1′-azobis(cyclohexanecarbonitrile) (ABCN)as the initiator, the hybrid polymer (polyMMA–MAPOM) could be prepared via the copolymerization of methyl methacrylate (MMA) and [N(C_4_H_9_)_4_]_3_[MnMo_6_O_18_{(OCH_2_)_3_CNH_2_}_2_](MAPOM). This ternary-resistance-switched memories device (ITO/PMMA–MAPOM/Pt) shows high tenability, with the number of effective charge carriers that could be well-tailored by the redox of the polyoxometalate under an electric field. The multi-redox states of Mn centers in the polyoxoanion {MnMo_6_O_18_} cluster enable ternary resistive switching behavior, exhibiting distinct ON, OFF, and intermediate conductive states. This could further influence the effective charge carrier densities of the hybrid-polymer switching layer. In another instance, the pyridyl organic terminus was introduced to chemically “solder” the POM cluster with two nanoelectrodes to investigate the charge transport through different oxidation states of the polyoxometalate.

#### 3.2.3. Stability

The ability to switch between the two resistive states at least 10^12^ times before irreversible state locking guarantees the lifetime of the memory can be in the order of years, which represents switching endurance.

The exceptional structural stability of polyoxometalates (POMs) is fundamental to memristor reliability, ensuring consistent redox activity and charge trapping for reproducible resistive switching. However, POM-based devices face limitations where degradation or agglomeration under electrical stress can cause parameter drift. While POMs on metal surfaces exhibit a novel insulator-to-metal transition via S–S bond formation—enabling switching without bulk structural change—challenges remain in enhancing long-term operational stability and ensuring CMOS compatibility for practical deployment [81] (As shown in Figure 4c). Stability in molecular electronic devices can be engineered through hierarchical integration of polyoxometalate (POM) clusters, spanning from precise single-molecule functionalization to controlled self-assembly of macroscopic cluster arrays. As an instance, Anderson–Evans polyoxometalate clusters with a capped pyridyl were also used to construct single-molecule junction devices via a covalent modification approach at a molecular level [116]. For instance, a pyridyl-terminated organic linker was strategically incorporated to chemically bridge the polyoxometalate (POM) cluster between two nanoelectrodes to investigate the charge transport through different oxidation states of the polyoxometalate. These single-molecule junction devices show a high-efficiency three-state transistor behavior enabled by their multiple charge/oxidation states and demonstrate the highly operational stability even under elevated voltage bias. These results demonstrate the strong potential of polyoxometalate (POM) clusters for advanced molecular electronics applications. The layer-by-layer (LBL) self-assembly approach has a high versatility in depositing ordered molecular layers of a Keggin cluster H_3_PW_12_O_40_ onto a 3-aminopropyl triethoxysilane (APTES)-modified silicon surface [100].

#### 3.2.4. Speed

A superfast crystallization speed can be realized by systematic analysis of discovered phase-change materials and ab initio elemental Sb-based molecular dynamics simulations are theoretically predicted to achieve a superfast crystallization speed. Improving the contact condition can be recognized as an approach to elevate the speed for POM-based memristors (As shown in Figure 4d) [117]. In the absence of solvent effect, the bridge oxygen of the polymolybdenum–oxygen cluster is more easily protonated than the counterpart of the end oxygen of the polytungsten–oxygen cluster (which is more likely to be protonated). The surface electrophilicity of polyoxometalate (POM) clusters governs their anionic charge states. The coordination numbers affect the charge transfer speed within the POM and thus the device speed. It can effectively regulate the reaction performance and realize coordination with multiple transition metal coordination ions. As an instance, by introducing a reducing agent, the metal center in the metal–oxygen cluster is reduced, leading to an enhancement of the nucleophilicity of its surface oxygen and the feasibility of organic–inorganic compounds to form polymetallic oxygen clusters with high coordination numbers [106,118].

The ON/OFF, storage density, and speed are all important factors to evaluate the performance of memristors and nonvolatile neuromorphic devices. The improvement of comprehensive properties is of great importance and is supposed to be given significant consideration. The current flow in molecular electronic systems is heavily reliant on molecule–electrode interfacial interactions, playing a key role in designing high-performance data storage devices. The mechanisms have been systematically investigated with an observation that contact geometry and the number of bonds between the redox-active molecules and the electrodes determine the current flow, involving the interaction mechanism between different electron configurations and alignment of the HOMO–LUMO gaps, energy levels Ef and contact condition [119].

## 4. The Intrinsic Multi-Level Switching, Electronic Properties of POMs and Their Memristive Behavior at Various Heterointerfaces

The working principle of resistive switching of POM-based memristor is the formation and rupture of the conductive filaments. In redox-based neuromorphic systems, a nanoscale redox reaction occurs to give rise to resistance change corresponding to an external electrical stimulus, which is highly appealing owing to their great potential to design next-generation neuromorphic computing systems 81 [84].

### 4.1. Intrinsic Multi-Level Switching and Tunable Electronic Properties of POMs

**Figure 5:**
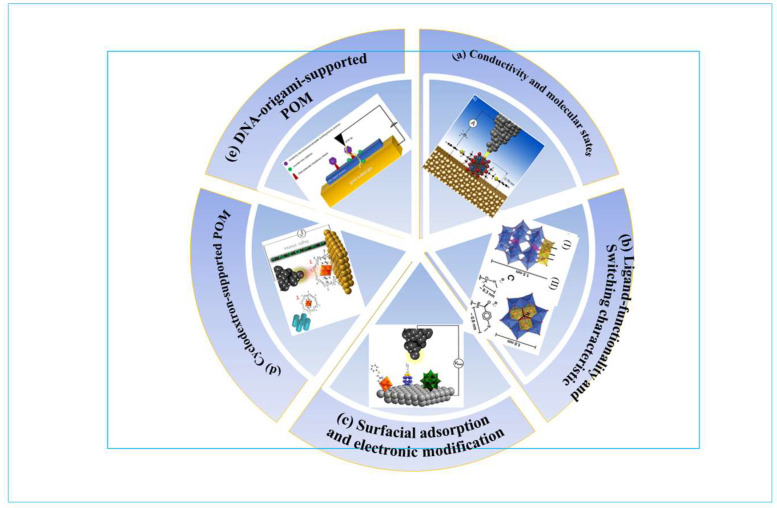
(**a**) Schematic representation of hexavanadate single-molecule 5 adsorbed on Au(111) with the above spotted STM tip [26]. Copyright: 2018, American Chemical Society. (**b**) The (OCH_2_)_3_CR ligated WD polyoxoanion. (I) Side view. (II) Top view. (III) Ligand scaffolds: R_1_ = CH_2_SMe of WDS-1 and R_2_ = NHCOC_6_H_4_SMe of WDS-2. Color code: blue octahedra = WO6, yellow octahedra = VO_6_, magenta square pyramids = PO_4_, red spheres = O. H atoms are not shown. Copyright 2022 Wiley VCH. (**c**) Adsorption and electron modification of polyoxometalates on surfaces [121]. Copyright: 2021 American Chemical Society. (**d**) Schematic of mass-selected Cyclodetrin-supported polyoxovanadates for multistate resistive switching memory applications [122]. Copyright: 2022 American Chemical Society. (**e**) Conceptual blueprint. “Metal−DNA-origami−polyoxovanadate” heterostructure enabling the implementation of memristive functions via multi-logic functions as offered by POV6 (color code: violet) and a synaptic dynamic as offered by the interplay between the gold surface (color code: dark yellow), DNA-origami (color code: blue), and counter ions (color code: green) [28]. Copyright: 2023 American Chemical Society.

Polyoxometalates (POMs) demonstrate a great many merits that make them exceptionally promising for next-generation molecular memristor and neuromorphic computing applications. The primary advantage lies in their ability to undergo multi-electron redox transformations without significant structural distortion [26,97]. This allows individual POM molecules to function as multi-bit switches, where discrete and stable conductivity states correspond to specific molecular redox states [26]. Scanning tunneling spectroscopy (STS) studies on Lindqvist-type hexavanadates have uncovered a Coulomb staircase-like behavior at room temperature, with each current step directly linked to the reduction in a single vanadium center within the cluster [26] (As shown in Figure 5a). This elucidated the relationships between the charge state of the molecule and its electrical conductivity, a fundamental requirement for multi-level resistive memories. Furthermore, the electronic structure of POMs, including their band gaps and the energetic position of their frontier orbitals, possesses high programmability by chemical modification. Particularly, the Wells–Dawson-type polyoxoanions exhibit a distinct ligand-functionality-dependent self-assembly behavior on surfaces; consequently, the alignment of their electronic band structure with the electrode’s Fermi level is tuned [120] (As shown in Figure 5b). This tunability is crucial for optimizing charge injection barriers and guaranteeing predictable device behavior. Moreover, computational studies have revealed that the electrical conductivity of adsorbed POMs is governed by the nature of the isolated molecule and that successive electron additions gradually increase conductivity, producing a staircase-like current–voltage dependency [121] (As shown in Figure 5c). This computational modeling provides strong evidence for understanding and predicting the performance of POM-based devices. The outstanding versatility of POMs is further demonstrated by their capabilities to form functional molecular devices with other functional molecules [122], as shown in Figure 5d or supramolecular structures [28], offering powerful synergistic advantages, as shown in Figure 5e.

These intrinsic electronic properties and the capability to support stable, well-defined multi-redox states endow POMs with a multi-bit switching characteristic. This provides a platform for sophisticated device functionalities: coupling electrical memory with visual readout, offering a unique advantage for state visualization in neuromorphic systems.

### 4.2. Multi-Modal Switching: Coupling Electrical Memory with Visual Readout in POMs

**Figure 6:**
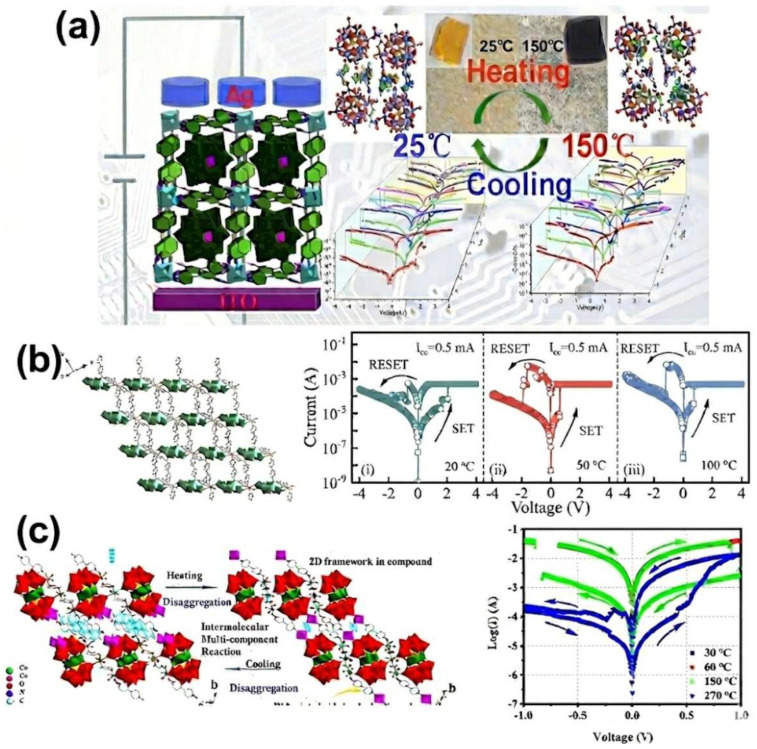
(**a**) (**Left**) panel: The structure of POMOF and its memory device. (**Right**) panel: the resistive switching performances at room temperature accompanied by thermochromism [90]. Copyright: 2021, American Chemical Society. (**b**) (**Left**) panel: The 2D layer of {NiII(1,4-cby)_2_[H_2_(γ Mo_8_O_26_)]}·4H_2_O. (**Right**) panel: I–V curves of ITO/POMOF/Ag devices at different temperatures of 20, 50, and 100 °C (Icc: 0.5 mA) [123]. Copyright 2023 American Chemical Society. (**c**) (**Left**) panel: Thermal-induced reversible structural and dimensional transformation between 1 and 2, accompanied by an intermolecular three-component aggregation or disaggregation process. WO_6_: red; PO4: yellow; Co_4_O16 core: green; [Co(H_2_O)_6_]^2+^: purple; bpdo guest: turquoise. (**Right**) panel: I–V characteristics of ITO/1/Ag device at different temperatures [124]. Copyright 2021 Wiley VCH.

Although POMs may not offer a decisive advantage in terms of switching ratio for memristors or neuromorphic computing devices, their distinctive redox properties enable a unique feature: a visible chromic response (e.g., color change) during resistive switching triggered by stimuli such as temperature. This intrinsic “visual electronics” capability presents a novel avenue for POMs in neuromorphic systems where operational state visualization is desirable. In 2021, thermochromic POM was conceptualized initially by being constructed from Keggin-type POMs and metalloviologen. As an example, the ITO/POM/Ag device exhibited excellent nonvolatile memory behavior with an ON/OFF ratio of 3.4 × 10^5^ and a set voltage (V_set_) of 1.4 V, maintaining functionality at high temperatures up to 150 °C. Particularly, its operational state at elevated temperatures could be directly monitored through a reversible thermochromic transition from yellow to black. Structural analyses revealed that the saturated Keggin-type [α-GeW_12_O_40_]^4−^ clusters were embedded within a [Co_2_(bpdo)_4_(H_2_O)_6_]_n_^4n+^ cationic framework, stabilized by multiple C–H⋯O_POM hydrogen bonds. At 150 °C, the loss of three lattice water molecules resulted in a more condensed structure, evidenced by shortened Ge/W/Co–O bond lengths, strengthened hydrogen bonds, and more distorted organic ligands. The switching mechanism was attributed to electron trapping by both the POM clusters and the metalloviologen units [90] (Figure 6a). Similarly, a 2D reversible thermochromic POM, {Ni^II^(1,4-cby)_2_[H_2_(γ-Mo_8_O_26_)]}·4H_2_O, has been designed with thermal stability and distinct temperature-modulated resistive switching. The device showed bipolar switching with an ON/OFF ratio of ~700, set/reset voltages of +1.2/−0.9 V, and tolerance up to 100°. Notably, the set voltage decreased while the reset voltage increased with rising temperature. The lower V_Set_ at high temperatures was explained by the thermally promoted formation of metallic conductive filaments (CFs) [123] (Figure 6b). However, the above works show that a relatively narrow electrochemical window observed in the I–V curve limits POMs’ broad application. In another study, a single-crystal-to-single-crystal (SCSC) transformation was induced by heat at 60 °C, where three isolated species aggregated into a 2D layer. This aggregation involved reversible structural adjustments, including dehydration/rehydration of Co-complexes, formation/cleavage of –bpdo–Co–bpdo–Co–bpdo– linkages, lattice shrinkage/expansion, and changes in structural dimensionality. As the temperature increased from 30 to 270 °C, the material underwent persistent, reversible structural transformations accompanied by multiple thermochromic changes. Most intriguingly, the device exhibited a temperature-triggered memory behavior: the ITO/POM/Ag structure was resistive-switching “silent” below 150 °C, but its memory performance could be “turned on” at 150 °C and maintained up to 270 °C [124] (Figure 6c).

### 4.3. Memristive Behaviors of POM at Various Heterointerfaces and Being Integrated with Other Materials

#### 4.3.1. Memristive Behaviors of POM at Various Heterointerfaces

The intrinsic multi-level switching and tunable electronic properties of POMs, along with the resulting competence for coupling electrical memory with visual readout, lay the foundation for integrating POMs with other functional materials. As a prerequisite to studying such hybrid systems, we first investigate the electrical behavior of POMs at semiconductor or electrode heterojunction interfaces. POM primarily forms heterojunctions on silicon surfaces and on gold surfaces.

**Figure 7:**
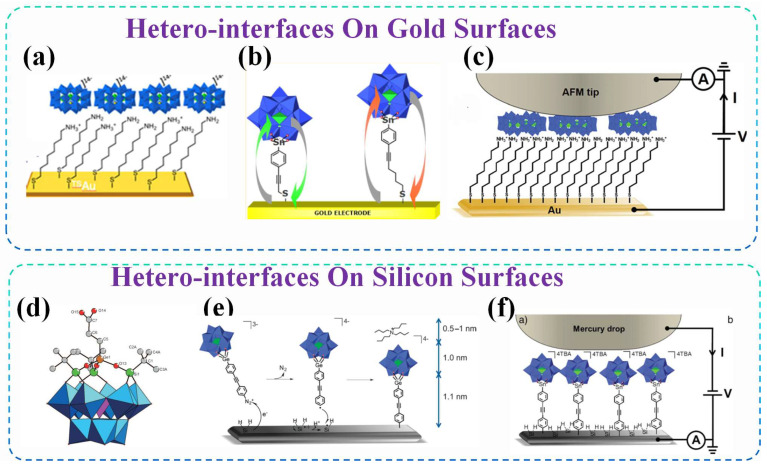
(**a**) Schematic description of the two-step fabrication of the self-assembled monolayers [125]. Copyright 2023 Royal Society of Chemistry. (**b**) Electron transfer to covalently immobilized Keggin polyoxotungstates on gold [126]. Copyright 2014 American Chemical Society. (**c**) Scheme of the molecular layer grafted on a Si substrate and the SMM junction Si-KW Sn//Hg [127]. Copyright: 2018 Royal Society of Chemistry. (**d**) Mixed polyhedral and ball-and-stick representation of [PW_9_O_34_(tBuSiO)_3_Ge (CH_2_)_2_CO_2_H]_3_ [128]. Copyright: 2010, Wiley-VCHt. (**e**) Schematic of grafting of diazonium post-functionalized POMs on a silicon surface [129]. Copyright 2015 Royal Society of Chemistry. (**f**) Scheme of the electronic transport characterization by C-AFM of the [H7P8W48O184]33− electrostatically deposited onto AOT SAM [130]. Copyright 2018 Royal Society of Chemistry. The capacity to precisely engineer POM–electrode interfaces, as demonstrated on silicon and gold, naturally extends to their integration with a wide range of nanomaterials, positioning POMs as a versatile platform for constructing next-generation hybrid devices.

On Gold Surfaces: POMs can also be constructed by functionalization with thiol-terminated linkers from stable self-assembled monolayers via Au–S bonding [125,126,127] (Figure 7a–c). These monolayers exhibit well-defined, surface-confined redox processes, and the kinetics of electron transfer from the gold electrode to the POMs can be precisely tuned by varying the length of the organic linker, with standard heterogeneous rate constants (k) ranging from 100 to 170 s^−1^ [126]. The relatively weak distance dependence observed for electron transfer to the POM centers suggests that the POM moieties may adopt a flattened orientation on the surface at negative potentials, facilitating efficient charge propagation [126]. Meanwhile, the utilization of ionic liquid electrolytes, such as [BMIM][BF_4_], endows POMs with stable electrochemical responses in these monolayers. Specifically, scanning electrochemical microscopy (SECM) reveals that well-organized POM monolayers demonstrate good lateral conductivity and the ability to reversibly accumulate negative charges—a key feature for charge-trapping memory devices [127]. Collectively, these studies underscore that, whether on silicon or gold, the precise control over the POM–electrode interface afforded by covalent or thiol-based anchoring is essential for harnessing their full potential in molecular-scale electronics.

On Silicon Surfaces: The covalent grafting of POMs onto hydrogenated silicon substrates facilitates the formation of dense, homogeneous monolayers with well-defined thickness and high packing density [128,129,130] (Figure 7d–f). This robust Si–C linkage ensures excellent mechanical and electrochemical stability, while preserving the intrinsic redox activity of the POMs [128]. Importantly, electron transport through silicon–POM–metal junctions is governed by the energy alignment between the silicon Fermi level and the lowest unoccupied molecular orbitals (LUMOs) of the POMs [130]. The measured tunneling barrier height (ΦPOM) directly reflects this alignment, and can be systematically modulated by changing the metal center within the POM framework (e.g., 1.8 eV for W-based POMs versus 1.6 eV for Mo-based POMs), correlating well with the trend in their solution-phase reduction potentials [130]. This retention of molecular identity in solid-state devices, combined with the compatibility with silicon-based technologies, positions POMs as highly tunable components for next-generation molecular electronics and memory applications [129,130].

#### 4.3.2. Integration with Other Materials

**Figure 8:**
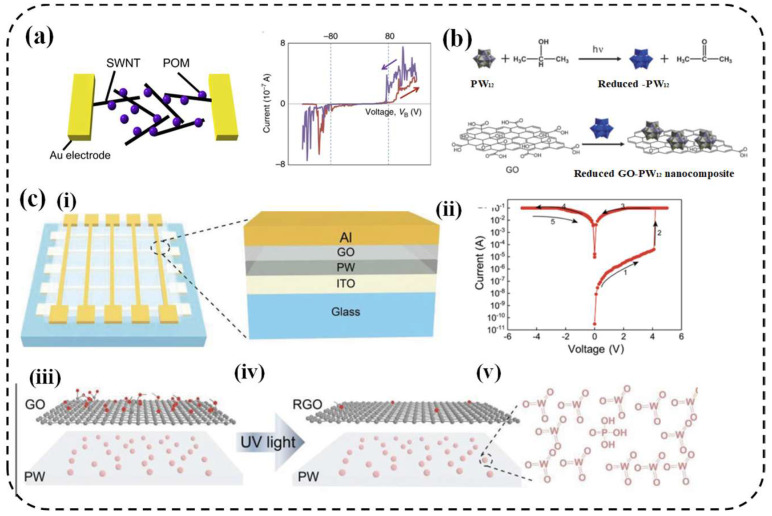
Integration of POM with other materials (**a**) Left panel: Schematic of a network with the single-walled carbon nanotubes SWNT/POM complex network. The yellow cuboids, black tubes and purple spheres represent the terminal electrodes, SWNTs and POM particles, respectively. Right panel: *I*–*V* curve of the water-treated POM/SWNT samples. A number of NDR peaks are noticeable over approximately 80 V [31]. Copyright 2018 Nature. (**b**) The mechanism of H_3_PW_12_O_40_-assisted photoreduction of graphene oxide [132]. Copyright: 2010 Royal Society of Chemistry. (**c**) (**i**) Schematic illustration of an arrayed and single sandwiched structural device (ITO bottom electrode/PW/PAH/GO/Al top electrode). (**ii**) The schematic illustration of the switching from self-assembled (**iii**) PW–GO to (**iv**) PW–RGO in the catalytic photoreduction process with UV illumination. (**v**) The molecular structure of the PW molecule [133]. Copyright 2019 Wiley VCH.

Owing to their structural versatility, POMs can be readily integrated with a wide range of materials, positioning them as a key multifunctional platform for constructing next-generation materials [131]. A molecular neuromorphic network based on a dynamic, ultra-dense assembly of single-walled carbon nanotubes (SWNTs) by incorporation of polyoxometalate (POM) molecules has been proposed. It is experimentally demonstrated that this SWNT/POM network exhibits spontaneous spiking activity and intrinsic noise—key characteristics of biological neural systems. The underlying dynamic mechanism can be elucidated by an electron-cascading model built on the network’s heterogeneous molecular junctions, the predictions of which align closely with our experimental observations. These findings suggest that complex, functional neuromorphic networks can be engineered from the bottom up using molecular-scale components, offering a promising pathway toward bridging the integration gap and advancing the development of high-density, brain-inspired computing devices (Figure 8a) [31]. To extend the application horizon of POM-based memristors, integrating or incorporating external materials is also a new tendency. Versatile compatibility facilitates POMs being incorporated into other materials (graphene oxide or reduced graphene oxide), particularly carton materials to enhance devices’ performance and functionality [132,133] (Figure 8b,c).

### 4.4. Comparative Performance Analysis of POM-Based Memristors and Metal Oxide-Based Memristors

Following the examination of their structures, mechanisms, and performance determinants, POMs and metal oxides are compared to assess their respective suitability for molecular neuromorphic devices. The metal oxide-based memristors, generally conforming to the valence change mechanism (VCM) and electrochemical metallization (ECM), have been a mature technology with large switching ratios but suffer from stochastic variability and high forming voltages. Addressing these limitations necessitates through molecular-level uniformity, unexceptional redox behavior, and structural tunability.

Leveraging the advantages of atomically precise, discrete molecular structures, polyoxometalates (POMs) are preferable to the conventional metal oxides for molecular devices. Unlike bulk or thin-film metal oxides, which suffer from stochastic variability, grain boundaries, and non-uniform defect distributions, POMs offer a well-defined platform where resistive switching originates from intrinsic, reversible redox reactions at specific metal centers (e.g., V, W, Mo) rather than from uncontrolled filament formation [6,7,8]. This molecular-level uniformity, demonstrated across Keggin [79,80,81,82], Wells–Dawson [83], and Lindqvist [25,31,32,33,34,84,85], and archetypes, enables highly reproducible multi-level resistance states via stepwise electron injection [26,120,122]. Particularly, their solubility and chemical versatility enable covalent or supramolecular functionalization with organic ligands [126,128,130], providing a pathway for constructing well-controlled self-assembly on electrodes (Au, Si) [125,126,127,128,129,130] and precise tuning of the energy level alignment relative to the Fermi level of the metal [120,130].

In summary, polyoxometalates have provided high reliability for neuromorphic engineering, attributed to their atomically precise structures and exceptional thermal/chemical stability that ensure reproducible resistive switching [25,75,76,77,78,84]. The multi-level data representation stems from underlying volatile resistance modulation, driven by stepwise metal reduction and coupled countercation dynamics. This mechanism favors both short-term memory with biologically relevant retention (50–300 ms) and long-term memory through controlled ion diffusion [28,29,30]—temporal dynamics fundamental to emulating synaptic plasticity [31,32]. In contrast to classical memory, where crosstalk is detrimental, POM-based networks intentionally leverage intermolecular communication via mobile countercations to support collective ionic interactions, enabling self-training and pattern classification [15]. Through strategic immobilization of either POMs or their countercations, three-dimensional neuronal networks can be constructed, effectively bridging molecular redox chemistry with advanced neuromorphic computing paradigms.

## 5. Challenge and Prospects

### 5.1. Challenge

Given the advantages of lower device-to-device discrepancies, robustness, solvent resistance, and huge storage ability, POMs provide unprecedented opportunities for developing next-generation advanced memristor or neuromorphic computing devices. The theoretical framework in the following aspects remains immature, which constrains the potential of POMs as next-generation advanced memristors or neuromorphic computing materials. Specifically, the theoretical architecture needs to be clarified from the following perspectives:

#### 5.1.1. Establish the Mechanism System on the Relationship Between Geometry and Operating Voltages

In polyoxometalate hybrid crystalline systems, interactions between the heterocyclic moiety and the cluster govern the device’s switching characteristics [134]. Geometry optimization plays a crucial role in enhancing device performance, as the molecular configuration directly influences the operating voltages. A higher voltage is required to write/erase, yet a plot of DVT versus logarithmic time demonstrates that the limit of the program/erase times and read times is relatively short. The modification of cluster electronic structures through heteroatom doping occurs via a well-defined redox mechanism. This can be explained by the fact that in this cluster type, the heteroatoms are perfectly positioned next to each other, enabling direct interaction through their lone electron pairs [106].

#### 5.1.2. Establish the Mechanism System on the Relationship Between Contact Geometry and Counter Ions and Charge Transfer

The contact geometry and counter ions critically govern current flow and charge transfer in POM-based devices or systems. At low drain bias, the primary transport pathway in the major POM-based molecular junctions is LUMO-dominated. The transmission characteristics are governed and modulated by the molecular electronic structure, interfacial geometry and ionic environment. Precisely, the contact geometry, combined with the number of bonds between the POM and the electrodes, determines the current flow [91]. In a molecular electronic system, apart from the intrinsic geometry, the current flow also heavily depends on the interaction of the molecule with the electrode contact. Comparative analysis of frontier orbital energetics demonstrates that molecule–electrode configuration predominantly determines the molecular energy level alignment, as evidenced by systematic variations in HOMO-LUMO gaps across the different junction geometries [135,136].

#### 5.1.3. Establish the Mechanism System on the Relationship Between Stacking Configuration and Storage Density

The advancements and rapid development of electronic information technology have created growing demands for information storage capacity, motivating the exploration of memristors/devices with a high data storage density. In addition to device miniaturization, building multilayer stacking structures can also be recognized as a rational strategy. In addition to scaling down, as well as the stacking structure approach, application of multi-level storage materials is the most rational strategy to achieve high-density memory devices [137,138]. The challenge demands cutting-edge technologies to garner information in terms of the diffusion and concentration of lattice oxygen ions and the valence state of metal ions on the nanoscale, which might lead to a deeper understanding and ways to control the redox reaction. Challenges also remain regarding storage speeds and low electrical conductivity, as well as compatibility for POM to be integrated into highly integrated and sophisticated POM-based neuromorphic devices. Strategies for optimal performance of POMs are listed as follows: The performance of POMs-based memristors can be attributed to three principal factors: (1) POMs exhibit reversible trap and detrapping behavior of a large number of electrons, with minimal structural reorganization. (2) Numerous polyoxometalate (POM) families exhibit multiple, well-defined redox states (typically 4–6 states) within a narrow electrochemical window (<0.5 V), indicating the capability of multibit data storage. (3) The delocalization of electrons over the POM skeleton, combined with the reorganization, exhibits a limited structure during the redox process, causing the long term stability of the programming/erasing cycle [18].

(1) The Introduction of Electronically Active Templates

Single-molecule electronic devices are engineered through the strategic incorporation of redox-active heteroatoms in Wells–Dawson cluster, resulting in distinct electrical properties of molecular devices compared to their bulk counterparts. The redox activity of POMs is high and can be doped with electronically active heteroatoms. The electronic and structural properties of the Wells–Dawson class endow the POM with applied non-volatile molecular memories [139].

(2) Structural Modification and Fabrication Optimization

The surface structure modulates electron transport of the memristor by influencing the electron cloud. The number of electrons that the POM has been accepting is determined by its charge-to-nuclearity ratio. Furthermore, in the case of hetero polyanions, the identity and oxidation state of heteroatoms are supposed to be taken into consideration [140].

### 5.2. Prospects

Leveraging the unique chromic response of polyoxometalates (POMs) to redox reactions, coupled with their excellent compatibility for hybridization with diverse matrices and molecular-level design precision that minimizes device discrepancies, POM-based materials present a promising avenue for the development of advanced neuromorphic devices and novel computing paradigms in artificial intelligence.

#### 5.2.1. Potential and Limitations of POMs in Intelligent Electronics and Neuromorphic Vision

**Figure 9:**
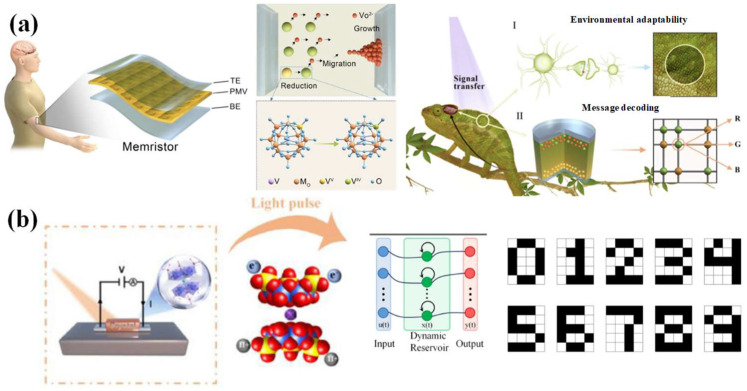
Advanced POM-based memristive and neuromorphic device applications. (**a**) Left panel: Schematic diagram of a bionic intelligent system using a PMV device. Middle panel: Schematic illustration of the resistive switching mechanism under electrical pulse stimulation. Right panel: Schematic representation of the biological responses (I) and information decoding (II) of the chameleon in various environments [141]. Copyright: 2025 American Chemical Society. (**b**) Left panel: Schematic diagram of a biological synapse and experimental setup of an optoelectronic memristor (the distance between the two electrodes is 50 μm). Middle panel: Schematic of a conceptual reservoir computing system. Right panel: Digital image containing various Arabic numerals [142]. Copyright 2025 Wiley VCH.

Owing to the characteristic of precise redox tunability and exceptional electron-accepting capability while maintaining remarkable structural stability, POMs represent molecular transition metal oxide clusters, exhibiting significant advantages over their bulk oxide counterparts. Responsiveness to multiple stimuli, ensuring stable and reversible color switching and resistive properties, makes them competent for various application scenarios. Recently, POMs have been revolutionarily employed in developing intelligent electronic devices that integrate memory and color-tuning capabilities [141] (as shown in Figure 9a). POM-based single-crystal also demonstrates powerful potential to bring transformative advancements in optoelectronic synapse. For example, 4-bit reservoir computing and 98.9% digit recognition accuracy via light-induced short-term plasticity have been achieved. While showcasing POMs’ potential for neuromorphic vision, the device critically relies on high humidity (98% RH) to enable proton-coupled electron transfer—failing to exhibit synaptic behavior at 25% RH. This humidity-dependent operation poses severe practical limitations for real-world deployment, where environmental stability is essential. Furthermore, single-crystal growth challenges and device scalability remain unaddressed, highlighting the gap between molecular design and practical hardware integration [142] (as shown in Figure 9b).

#### 5.2.2. Nanometer and Functionalization Development

A key research focus in polyoxometalate (POM) nanotechnology involves the rational design of nanostructured POMs to improve their application performance in different fields by regulating particle size, morphology, and surface structure. In addition, the storage capabilities (high-performance memristor possessing high ON/OFF (above 10^6^)), good durability are further improved by means of ligand modification and heteroatom doping [143,144,145,146].

#### 5.2.3. Extension of Electrochemical Applications

Building on their established success in electrochemical energy systems (lithium/sodium-ion batteries and supercapacitors), polyoxometalates (POMs) now show exceptional promise for developing integrated self-powered neuromorphic computing systems (constructing an electrochemical neuromorphic computing transistor with an electrochemical impedance of less than 0.1 MΩ, a conversion frequency range from 10 mHz to 5 MHz, and a synaptic weight exceeding 120%) [115].

## 6. Conclusions

To summarize, this review offers comprehensive information and substantial references for exploring and advancing next-generation POM-based electronic devices. To summarize, this review gives a systematic examination of the fundamental knowledge of POMs, encompassing the memristor mechanism and the classification, applications, parameters, and typical instances of POM materials, and it highlights the advantages of polyoxometalate (POM) in memristors/neuromorphic system applications. Particularly, the guidance for designing POM-based devices is presented by outlining performance evaluations and corresponding strategies for improving performance evaluations. Encompassing both foundational principles and recent breakthroughs in POM neurocomputing, this study further contributes practical suggestions for system optimization and potential improvements. In all, this review offers comprehensive information and substantial references for exploring and advancing next-generation POM-based electronic devices.

## Figures and Tables

**Table 1:** A Comparison of the three structural classes of polyoxometalates.

Materials	Switching	Vset/Vreset	ON/OFF	Endurance	Multi-Level Behavior	Variability	Ref
Keggin type	Bipolar	−7~0.7 V	1.2 × 10^4^–7 × 10^3^	>10^3^ cycle	Executable	medium	[79,80,81,82]
Dawson-type	Bipolar	~−2~4 V	1.18 × 10^3^–2.7 × 10^4^	>10^3^ cycle	Executable	medium~excellent	[83]
Lindqvist-type	Bipolar	~−0.5~2 V	10–10^3^	>10^3^ cycle	Executable	medium	[25,31,32,33,34,84,85]

## Data Availability

No new data were created or analyzed in this study.

## Abbreviations

The following abbreviations are used in this manuscript:

POM	polyoxometalates
GO	graphene oxide
COMS	Complementary Metal Oxide Semiconductor
